# Effect of Feeding Acid Oils on European Seabass Fillet Lipid Composition, Oxidative Stability, Color, and Sensory Acceptance

**DOI:** 10.1155/2023/6415693

**Published:** 2023-01-06

**Authors:** Paula Albendea, Alba Tres, Magdalena Rafecas, Stefania Vichi, Roser Sala, Francesc Guardiola

**Affiliations:** ^1^Nutrition, Food Science and Gastronomy Department-XIA, Campus de l'Alimentació Torribera, Facultat de Farmàcia i Ciències de l'Alimentació, Universitat de Barcelona, Av Prat de la Riba, 171., 08921 Santa Coloma de Gramenet, Spain; ^2^Institut de Recerca en Nutrició i Seguretat Alimentària (INSA-UB), Universitat de Barcelona, Av Prat de la Riba, 171., 08921 Santa Coloma de Gramenet, Spain; ^3^Nutrition, Food Science and Gastronomy Department-XIA, Facultat de Farmàcia i Ciències de l'Alimentació, Universitat de Barcelona, Av Joan XXIII, 27-31., 08028 Barcelona, Spain; ^4^Animal Nutrition and Welfare Service (SNiBA), Animal and Food Science Department, Facultat de Veterinària, Universitat Autònoma de Barcelona, Travessera dels Turons., 08193 Bellaterra, Spain

## Abstract

Acid oils (AO) are fat by-products of edible oil refining with a high energetic value, being an interesting option for a more sustainable aquaculture nutrition. This study was conducted to evaluate the effects of the partial replacement of fish oil (FO) in diets by two AO instead of crude vegetable oils on the lipid composition, lipid oxidation and quality of fresh European seabass fillets, and after their commercial refrigerated storage for 6 days. Fish were fed with five different diets, the added fat being FO (100%) or a blend of FO (25%) and another fat (75%): crude soybean oil (SO), soybean-sunflower acid oil (SAO), crude olive pomace oil (OPO), or olive pomace acid oil (OPAO). Fresh and refrigerated fillets were assessed for fatty acid profile, tocopherol (T) and tocotrienol (T3) composition, lipid oxidative stability, 2-thiobarbituric acid (TBA) value, volatile compound content, color, and sensory acceptance. Refrigerated storage did not affect T + T3 total content but increased secondary oxidation products (TBA values and volatile compound contents) in fillets from all diets. The FO substitution decreased EPA and DHA and increased T and T3 in fish fillets, but the recommended human daily intake of EPA plus DHA could still be covered with 100 g of fish fillets. Both a higher oxidative stability and a lower TBA value were found in SO, SAO, OPO, or OPAO fillets, obtaining the greatest oxidative stability in OPO and OPAO fillets. Sensory acceptance was not affected by the diet or the refrigerated storage, while the differences found in color parameters would not be perceived by the human eye. According to the oxidative stability and acceptability of flesh, SAO and OPAO are adequate replacements of FO as energy source in European seabass diets, which implies that these by-products can be upcycled, improving the environmental and economical sustainability of aquaculture production.

## 1. Introduction

As the years have gone, the importance of aquaculture production to cover food fish demand has significantly raised [1]. This increasing trend is expected to continue in the future, reaching to cover 62% of food fish production in 2030 [2]. In 2018, 88% of fish production was intended for human consumption while the rest had other uses, as fish oil (FO) and fish meal production [1]. The commonest lipid source used in fish feed has been FO due to its high percentage of n-3 long-chain polyunsaturated fatty acids (n-3 LC-PUFA), such as eicosapentaenoic acid (C20:5 n-3, EPA) and docosahexaenoic acid (C22:6 n-3, DHA), which are considered essential fatty acids (FA) for fish [3, 4].

The increase of aquaculture production has implied a raise in the demand of FO for fish feeding, while the production of FO has remained stable. As this could jeopardize aquaculture sustainability, it has been very important to find alternative fat sources to partially replace FO in fish diets. This substitution should guarantee enough EPA and DHA for an adequate growth and development of fish, especially for marine species such as European seabass (*Dicentrarchus labrax*) [5, 6]. Moreover, EPA and DHA content in fish diet influences their content in fish flesh, and, since these FA have shown health benefits, it also affects the nutritional value of fish flesh [7]. On the other hand, EPA and DHA have a great tendency to suffer oxidation reactions due to their high number of double bonds, and, consequently, oxidative rancidity is one of the main nonmicrobiological reasons of fish and fish food spoilage [8].

The options that have drawn more attention to replace FO as energy source are vegetable fat sources, the most common being soybean, linseed, rapeseed, sunflower, palm, and olive oil [6]. These fats have low EPA and DHA percentages but high percentages of C18 polyunsaturated fatty acids (PUFA) or monounsaturated fatty acids (MUFA) [9]. Also, some vegetable fats are usually characterized for being rich in tocopherols (T), tocotrienols (T3), and other bioactive compounds, such as polyphenols [9]. Thus, introducing vegetable fat sources to partially replace FO in fish feeding might cause both a FA profile modification of fish flesh, leading to a reduction of its EPA and DHA contents, and an increment in antioxidant compounds in fish flesh, which could prevent the development of lipid oxidation reactions and enhance its preservation [10].

Although crude oils are the most widely used vegetable sources to replace FO, it is also possible to replace it with some fat by-products from the food industry such as acid oils (AO). AO are fat by-products coming from edible oil refining, characterized for having a similar FA profile to the crude oil and a high content of free fatty acids (FFA), as they are obtained from the steps in which FFA are removed from the crude oil [11]. Using AO as feed fats alternatively to other lipid sources is a way to upcycle them, which would contribute to improving aquaculture sustainability. However, there are only a few trials performed with these by-products in the literature, focusing mainly on their effects on lipid digestibility and productive parameters and showing controversial results [12, 13]. In fact, as described by Varona et al. [11, 14], one disadvantage of AO is their high variability in various compositional parameters such as FFA content, insoluble impurities (I), or unsaponifiable matter (U). This affects their nutritional value [11], and consequently, this could impair the animal performance, decreasing the confidence of producers in these feeding fats. Also, the fact that the content of certain antioxidants, such as T and T3, is highly variable in these refining by-products could affect the oxidative stability and the quality of fish fillets during their commercial shelf life [11, 14]. However, the effects of using AO in fish diets on the composition and quality of fish flesh and fish food products are barely studied, which would be essential to upcycle them assuring the nutritional value, oxidative stability, and sensory quality of the fish products even after their storage under commercial conditions.

The aim of this work was to evaluate the effect of the partial replacement of FO in fish diets by AO instead of crude oils from two different vegetable sources (soybean and olive pomace) on lipid composition, oxidative stability, color, and sensory acceptance of fresh and refrigerated European seabass fillets. This study complements the information published by Verge-Mèrida et al. [13] on growth and digestibility parameters of the same specimens used in the present work.

## 2. Materials and Methods

### 2.1. Experimental Fats

Five different experimental fats were used: fish oil (FO), two crude vegetable oils with different FA profile, crude soybean oil (SO) and crude olive pomace oil (OPO), and two AO available in the Spanish market with similar FA composition to theirs, soybean-sunflower (55 : 45, w/w) acid oil (SAO) and olive pomace acid oil (OPAO) (Table 1). Bunge Ibérica S.A.U. (Sant Just Desvern, Spain) supplied SO and SAO, General d'Olis i Derivats S.L. (Les Borges Blanques, Spain) provided OPO, RIOSA (Refinación Industrial Oleícola S.A., Ibros, Spain) supplied OPAO, and AFAMSA (Agrupación de Fabricantes de Aceites Marinos S.A., Mos, Spain) supplied the commercial degummed FO, which mostly came from tuna.

The contents of moisture and volatile matter (M), I, U, T and T3, and FA and lipid class compositions, and acid and peroxide values of these five fat sources (Table 1) were determined by triplicate as described by Varona et al. [15].

### 2.2. Animals and Diets

The animal housing and husbandry was conducted at the Aquaculture Center facilities of the Institute of Agrifood Research and Technology (IRTA, Sant Carles de la Ràpita, Spain). All the procedures were approved by the Animal Protocol Review Committee of the Universitat Autònoma de Barcelona (UAB) and were in accordance with the guidelines set by the EU Directive 2010/63/EU [16]. A total of 300 European seabass (*Dicentrarchus labrax*) specimens of approximately 100 g were randomly distributed into 15 tanks of 500 L of capacity (20 fish per tank) with a sea water semirecirculation system (IRTAmar®; IRTA, Sant Carles de la Ràpita, Spain). This system allowed water recirculation of between 1 and 1.5 tank volumes per hour (15 m^3^/h) and was equipped with an aerobic biofilter for the removal/transformation of ammonia to nitrite and nitrite to nitrate. The fish were reared indoors and subjected to natural photoperiod and breed under controlled conditions of water temperature (22.6 ± 0.8°C), dissolved oxygen levels (7.3 ± 0.7 mg/L), pH (7.7-8.1), and salinity (35.5 ± 0.5%).

The experimental diets were prepared in form of extruded pellets by the Service of Experimental Diets of the University of Almería (Almería, Spain) using standard aquafeed procedures. The ingredients and the proximate composition of the diets are presented in Table 2. All the diets contained a total of 15.4% (w/w) added fat. In the control diet, the added fat source was 100% FO. In the other diets, a 75% of the added fat was one of the vegetable fat sources (SO, SAO, OPO, or OPAO), and the remaining 25% was FO to guarantee enough quantity of essential FA for European seabass (at least 1% of EPA + DHA on dry weight [17]). Feed samples were taken, and the FA composition, T and T3 content, and lipid class composition were determined in duplicate in all diets (Table 3). Therefore, considering the M and the FA profile of each diet (Tables 2 and 3), the EPA + DHA content was expressed on dry weight, being 5.9% in FO diet and 1.8% in the rest of diets. Thus, in all cases, EPA + DHA content was higher than the 1% of recommended for European seabass [17].

The five diets were randomly assigned to different tanks (three tanks per experimental diet), and the experimental feeding period lasted for 100 days. The effects of these diets on animal performance parameters have been published elsewhere [13].

### 2.3. Sampling of European Seabass Fillets

At the end of the experimental period, fish were fasted for 24 h and slaughtered by immersion in ice-cold water (hypothermia). Five replicates per dietary treatment were prepared. Each replicate was composed by six different fish, obtained from 3 tanks (2 fish from each tank) fed with the same diet. From each fish, the two entire fillets without skin were taken as samples, so a total of twelve fillets (3 tanks per diet × 2 fish per tank × 2 entire fillets per fish) were used for one replicate. All the samples were kept in zipper bags on ice until the next morning.

In less than 24 h after slaughter, the twelve fillets per replicate were divided into two groups of six fillets (one fillet of each fish per group), using one group to constitute the fresh sample and the other group to constitute the refrigerated sample. For fresh samples, the six fillets of each replicate were pooled and ground. After measuring the color, various aliquots of 25 g of the ground sample were immediately vacuum packed in high-barrier multilayer bags (Cryovac BB3255; permeability to O_2_, 17 cm^3^/m^2^ per day per bar at 23°C and 0% relative humidity, ASTMD-3985; Cryovac Europe, Sealed Air S.L., Sant Boi de Llobregat, Spain) and were kept at -20°C until the chemical analyses were performed. The other part of the ground sample was pooled with the ground sample remains from other replicates of the same diet and used to make hamburgers, which were cooked to perform the sensory acceptance test.

For refrigerated samples, the six fillets of each replicate were stored for 6 days under commercial conditions (at 2°C, packed in a modified atmosphere of CO_2_/N_2_/O_2_, 40/30/30). After the refrigerated storage, fillets were pooled and ground. The color determination, the sampling for chemical analysis, and the sensory acceptance test were done as detailed for fresh samples.

### 2.4. Determination of Fatty Acid Composition

The FA composition of fresh fish fillets (1.5-1.6 g) was analyzed by gas chromatography with flame ionization detector (GC-FID) after extracting the lipid fraction with chloroform/methanol (2 : 1, v/v) mixture. First, 20 mL of this mixture was added to the sample and homogenized using a high-speed homogenizer (PT 3100 Polytron, Kinematica, Lucerne, Switzerland) at 19,000 rpm for 20 s and filtered through Whatman No. 1 filter paper. The sample residue retained in the filter was reextracted twice with 10 mL of the same solvent mixture at 19,000 rpm for 20 s. Next steps of lipid extraction were carried out as described by Bou et al. [18]. Then, FA methyl esters were obtained from the extracted lipid fraction by a double methylation procedure and determined by GC-FID [15]. Each compound was identified by comparing its retention time with that of standards (Supelco 37 component FAME Mix, Supelco®, Merck KGaA, Darmstadt, Germany). The percentage of each FA was obtained by peak area normalization.

### 2.5. Determination of Tocopherol and Tocotrienol Content

For the determination of the T and T3 content in fresh and refrigerated fish fillets, 2 g of ground sample was homogenized with a mixture of antioxidants in ethanol using a PT 3100 Polytron for 30 s at 20,000 rpm and saponified with methanolic KOH as described by Bou et al. [18]. The nonsaponifiable fraction was extracted with petroleum ether. The solvent was evaporated under a nitrogen stream at 30°C, and the residue was dissolved in 99% n-hexane and injected into the chromatographic system. High performance liquid chromatography separation was carried out as described by Aleman et al. [19], and T and T3 were detected using a 1260 Infinity II Fluorescence Detector (Agilent Technologies, Santa Clara, CA, USA), setting the excitation and emission wavelengths at 290 and 320 nm, respectively. Each sample was analyzed twice, and calibration curves were prepared using *α*-, *β*-, *γ*-, and *δ*-T standards (Calbiochem®, Merck KGaA, Darmstadt, Germany). The content of *α*-, *β*-, *γ*-, and *δ*-T3 was calculated by applying the calibration curve obtained for the corresponding T analogue.

### 2.6. Ferrous Oxidation-Xylenol Orange (FOX) Method

The FOX method was applied in each sample per triplicate to evaluate the primary oxidation and the oxidative stability of fresh and refrigerated fillets, as detailed by Grau et al. [20]. Briefly, 15 mL of cold methanol was added to 2 g of sample, and a PT 3100 Polytron was used for sample homogenization at 12,000 rpm for 30 s. Then, the mixture was centrifuged (1,400 g, 3 min), and the supernatant methanol extract was collected. The reaction mixture was prepared in glass cuvettes, capped with Teflon caps. The reagents were added as described by Tres et al. [21], using 1030 *μ*L of methanol and 70 *μ*L of sample extract. The absorbance at 560 nm was measured using a UV-3600 spectrophotometer (Shimadzu, Kyoto, Japan) after incubation for 30 min (as a measure of the lipid hydroperoxides (LHP) present in the samples from the beginning, named LHP content) and for 96 h (as a measure of the amount of LHP formed during this time which is considered a measure of sample's oxidative stability, named final LHP value). LHP concentration was expressed as mmol of cumene hydroperoxide (CHP) equivalents/kg of sample, with reference to a calibration curve prepared using CHP as standard (technical grade, 80%, Sigma-Aldrich®, Merck KGaA, Darmstadt, Germany).

### 2.7. Determination of 2-Thiobarbituric Acid (TBA) Value

The 2-thiobarbituric acid (TBA) value was determined twice per sample to evaluate the secondary oxidation of fresh and refrigerated fillets, applying the method described by Grau et al. [22] on 1.5 g of sample. The TBA value was measured through third derivative spectrophotometry after an acid aqueous extraction. The results were expressed as malondialdehyde (MDA) concentration (*μ*g/kg), using a calibration curve prepared as described by Botsoglou et al. [23] with 1,1,3,3-tetraethoxypropane (Sigma-Aldrich®, Merck KGaA, Darmstadt, Germany) as MDA precursor.

### 2.8. Determination of Volatile Compound Content

The volatile compound content was determined in fresh and refrigerated fillets by headspace solid-phase microextraction coupled with gas chromatography and mass spectrometry (HS-SPME-GC-MS). To perform the analysis, 1 g of sample was weighed into a 10 mL screw-capped vial, and 0.5 mL of a 4 mg/L aqueous solution of 4-methyl-2-pentanol (97%, Sigma-Aldrich®, Merck KGaA, Darmstadt, Germany) was added as internal standard. Subsequently, 0.5 mL of an aqueous antioxidant solution with 4% of EDTA and 0.4% of propyl gallate (both from Sigma-Aldrich®, Merck KGaA, Darmstadt, Germany), 2 mL of double deionized water, and three glass balls were added. The vial was immediately closed and kept in ice until all sample set was prepared. Then, the mixture was homogenized using an ultrasound bath at 4°C for 10 min. Samples were kept in ice at the dark until the HS-SPME-GC-MS determination was carried out. The instrument consisted of an Agilent 6890N Network GC system with an Agilent 5975C Inert MSD quadrupole mass spectrometer (both from Agilent Technologies Santa Clara, CA, USA) and a PAL autosampler (CTC Analytics, Zwingen, Switzerland) configured to perform SPME. After 10 min of sample conditioning at the extraction temperature (45°C), the fiber of divinylbenzene/carboxen/polydimethylsiloxane (2 cm length, 50/30 thickness) from Supelco® (Merck KGaA, Darmstadt, Germany) was exposed to the head space for 30 min and desorbed in the injector at 260°C for 10 min. To perform the separation of the different volatile compounds, a Supelcowax-10 capillary column (30 m × 0.25 mm i.d., 0.25 *μ*m film thickness) from Supelco® (Merck KGaA, Darmstadt, Germany) was used. The oven temperature program began at 40°C (held 10 min, during fiber desorption time), 3°C/min up to 150°C, and 15°C/min up to 250°C (held for 5 min). Helium was used as gas carrier with a constant flow of 1 mL/min. The temperatures of the ion source and the transfer line were 230 and 280°C, respectively, and the ionization energy was 70 eV. Data were acquired in full scan mode in selected representative samples for the identification of compounds, which was carried out by comparison of their mass spectra and retention times with those of standard compounds or with those available in mass spectrum library Wiley 6 and in the literature. Then, the quantitative assessment of all samples was carried by selected ion mode, considering m/z 44, 45, 55, 56, 57, 58, 70, 81, and 98, which were representative for the compounds of interest. Data were then analyzed by an Agilent MSD ChemStation. Relative amounts of volatile compounds were calculated by the internal standard method, expressing the results as *μ*g of 4-methyl-2-pentanol equivalents/kg of sample.

### 2.9. Color Determination

Immediately after grinding the fillet samples, color was measured by CR-410 Konica Minolta colorimeter (Tokyo, Japan) based on CIE L^∗^a^∗^b^∗^ color space. The color parameters determined were lightness (L^∗^), from dark (0) to light (100); redness (a^∗^), from green (–a^∗^) to red (+a^∗^); and yellowness, (b^∗^) from blue (–b^∗^) to yellow (+b^∗^), as recommended by the International Commission on Illumination in 1976 [24]. The instrument was set for D-65 illuminant at a 2° observer angle and calibrated prior to the determinations with a standard white plate. Five measures were taken for each replicate in random different locations of the ground samples, and the average L^∗^, a^∗^, and b^∗^ was calculated.

The dimensionless parameter Δ*E* [25] was used to evaluate if the differences in color parameters between different sample groups could be perceptible by the human eye.

(1) $$\begin{array}{c} ∆E=\sqrt{∆{L^{*}}^{2}+∆{a^{*}}^{2}+∆{b^{*}}^{2}.} \end{array}$$

In this study, two different Δ*E* parameters were obtained:

- Δ*E*_*R*_ was calculated for each dietary treatment to evaluate the differences between the color parameters of fresh and refrigerated fillets. In this case, Δ*L*^∗^, Δ*a*^∗^, and Δ*b*^∗^ in Equation (1) corresponded to the difference between the L^∗^, a^∗^, or b^∗^ means of the fresh samples (*n* = 5) and of the refrigerated samples (*n* = 5) of the same dietary treatment
- Δ*E*_*D*_ was used to study the differences in color parameters of fresh or refrigerated fillets between the control diet (FO) and the other four dietary treatments. Thus, two different Δ*E*_*D*_ were obtained for each dietary treatment: one for fresh fillets and another for refrigerated fillets. In this case, the Δ*L*^∗^, Δ*a*^∗^, and Δ*b*^∗^ in Equation (1) corresponded to the difference between L^∗^, a^∗^, or b^∗^ means of fresh (*n* = 5) or refrigerated samples (*n* = 5) from FO diet and those from each one of the other four dietary treatments

A value of Δ*E* > 5 [26] was considered as the cut-off value above which the instrumental color differences would be perceived by the human eye.

### 2.10. Sensory Acceptance Test

Two nine-scale hedonic tests were performed to evaluate if there were significant differences in overall acceptance between the dietary treatments in fresh or refrigerated fish fillets. In both cases, hamburgers with 22.5 g ground fish fillet per unit were prepared and cooked for 3 min using five different machines (model SS-5515 750 W, Jocca, Zaragoza, Spain), one for each dietary treatment. A total of 30 regular fish consumers participated in each test. In each test, each participant evaluated five hamburgers (one per dietary treatment) and, for each of them, indicated the degree of acceptability on a 9-point scale (1: “dislike extremely”; 5: “neither like nor dislike”; 9: “like extremely”).

### 2.11. Statistical Analysis

Statistical tests were carried out with SPSS (version 20.0, IBM Statistics Inc.). Multifactor ANOVA (SPSS GLM procedure) was used to study the influence of the refrigeration time (0 and 6 days) as one main factor and its interaction with the main factor dietary treatment (FO, SO, SAO, OPO, or OPAO) on all the parameters evaluated in fish fillets (*n* = 50, 2 refrigeration times × 5 dietary treatments × 5 replicates each; for sensory acceptance *n* = 300, 2 refrigeration times × 5 dietary treatments × 30 replicates each), except for FA profile, which was determined only in fresh samples. One-way ANOVA (SPSS GLM procedure) was used to assess if there was a significant influence of the dietary treatment (FO, SO, SAO, OPO, and OPAO) on all the parameters studied in fresh fillets (*n* = 25, 5 dietary treatments × 5 replicates each; for sensory acceptance *n* = 150, 5 dietary treatments × 30 replicates each) and in refrigerated fillets (*n* = 25, 5 dietary treatments × 5 replicates each; for sensory acceptance *n* = 150, 5 dietary treatments × 30 replicates each). Significant differences among dietary treatments found by one-way ANOVA in fresh or refrigerated fillets were evaluated by the Scheffé's post hoc test (SPSS GLM procedure). Pearson's correlation test was performed to study the correlations of the oxidative parameters determined in fillets with the FA profile (*n* = 25) and between all the parameters analyzed in fresh and refrigerated fillets (*n* = 50). In all cases, differences were considered significant when *p* < 0.05.

## 3. Results and Discussion

As previously reported, AO present different compositional traits than crude oils and a high variability in their composition [11, 14], and since this can affect their nutritional value, it is important to properly characterize them to evaluate their quality as feed fats. The two AO assayed in this study (SAO and OPAO) presented a higher FFA content than SO and OPO (Table 1), as they are refining by-products coming from neutralization, a refining step performed to remove FFA from crude fats. These FFA percentages were in agreement with the ones reported by Varona et al. [11] for various AO. Another characteristic of AO is that they also tend to accumulate substances present in the I and U fractions, increasing their total MIU value [11]. The total MIU value is a parameter commonly used to evaluate the quality of feed fats as it represents the content of substances that can dilute their energy value. Accordingly, the total MIU values of the two AO used in this study were higher than those of the rest of the fat sources, with the percentages of M, I, and U, and total MIU being similar or lower than the usual levels found for these type of AO in Spanish market [11]. For both SAO and OPAO, U was the fraction with the greatest contribution to the total MIU value (Table 1). Remarkably, the recommendations published by FEDNA [27] for the use of AO in animal diets establish a total MIU value lower than 5%, which was fulfilled by the SAO and slightly exceeded by OPAO. In fact, in the performance parameters resulting from the experimental diets used in this work and that were reported by Verge-Mèrida et al. [13], no effect was observed when SAO was used, but a significantly lower performance was found when fish were fed with OPAO diet, showing the lowest weight (226.22 g vs. the range of 244.45–250.20 g), specific growth rate (0.80%/day vs. the range of 0.88–0.90%/day), and average daily gain (1.25 g vs. the range of 1.43–1.49 g). Regarding the apparent digestibility of dry matter, crude protein, or total FA, no effect was observed when AO were included in the diets [13]. Beyond this, to promote the upcycling of this type of refining by-products as vegetable feed fats to replace FO for aquaculture nutrition, it is important to comprehensively study their impact not only on fish performance parameters but also on the composition, oxidative stability, and sensory acceptability of the flesh, so that the interest of the various stakeholders (feed producers, farmers, and consumers) is addressed.

### 3.1. Fatty Acid Profile of Fish Fillets

The results of the FA profile of fresh fillets coming from each diet are presented in Table 4 (the complete FA composition including minor FA can be found in Table S1 in supplementary material).

The differences in the FA profile of fish fillets between dietary treatments were significant for all the determined FA and for the sums of each FA type. Most of the trials found in the literature showed that the FA profile of fish fillets mirrored the FA composition of the diet [6, 28]. In our study, fillets coming from FO diet had the highest content in EPA (5%) and DHA (16%), whereas the other four diets led to ≈3% of EPA and to similar levels of DHA (≈8%) in fillets (Table 4). This was in agreement with the EPA and DHA reductions observed in the diets when FO was partially substituted by vegetable sources (from 7% of EPA and 22% of DHA in the FO diet, to approximately 2% of EPA and 7% of DHA in the rest of diets) (Table 3). Similarly, previous studies reported that the main effect of the partial or total substitution of FO with vegetable oils is a reduction of EPA and DHA content in the whole fish, organs, and flesh [10]. However, considering that the general recommendation for the daily EPA plus DHA intake is 250 mg [29] and taking into account the amount of fat extracted from fish fillets in this study (data not shown), 100 g of FO fillets would cover 302% of the recommended EPA plus DHA daily intake, whereas fillets from the other four diets would cover between 168% and 192%. Therefore, even though FO was partially replaced with the AO or crude oils, the content of these FA in fish fillets was enough to cover the recommended EPA + DHA daily intake. Nevertheless, it is important to remind that these LC-PUFA are highly prone to lipid oxidation, and, as it will be commented below, they might influence the oxidative stability of fish fillets.

The decrease in saturated fatty acid (SFA) percentage (from 27% to 22%–23%) in fish fillets observed with the partial substitution of FO by SO, SAO, OPO, or OPAO was related to the FA profile of the feed fats and of the diets, as FO diet showed the highest content in SFA (35%). This was also in concordance with previous trials. For example, Mourente and Bell [5] also found a reduction of SFA levels in European seabass fillets when a 60% of FO was replaced by different blends of vegetable fat sources in fish diets with 16% added fat. However, the decrease in the SFA percentage (from 24% to ≈21%) was lower than the one observed in this study, as their vegetable blends included palm oil. Another work representative of this fact is the one carried out by Izquierdo et al. [30] who, despite of working with another species (gilthead seabream) and of replacing FO by 60% or 80% of different vegetable oils (linseed, rapeseed, or S) in diets with 17.6% added fat, observed a SFA reduction in fillets (from 28% to the range of 24%–21%) similar to the one found in this study.

Other distinctive aspects of the FA profile of the fat sources and diets were also reflected in the FA composition of fillets (Table 4). For example, fillets from fish fed with SO diet showed the highest levels of linoleic (26%) and linolenic (4%) acids. In general terms, the FA profiles of SO and SAO fillets were similar, although differences were found for oleic (C18:1 n-9) and linoleic (C18:2 n-6) acids. Again, these differences reflected the ones observed between the corresponding diets (Table 3) and between the two fats (SO and SAO) (Table 1). In fact, the lower linolenic acid content in SAO compared with SO evidenced that this fat by-product contained AO from the refining of soybean and sunflower oils [31]. Previous works performed using diets formulated with SO as a partial replacement of FO also revealed increments of linoleic and linolenic acids in fish muscle. For example, Izquierdo et al. [32] observed that linoleic acid content in seabass muscle raised from 5% to 17% and linolenic from 1% to 2% when using a diet with 19.7% of added fat and a 60% replacement of FO by SO. The lower linoleic acid percentage and the lower increase found by them in comparison to our results can be explained by the presence of a lower content of this FA in their diets (7% in FO and 26% in SO). The same work showed an influence of the fish species on the tendency to mirror the FA profile of the diet, with the increase of the linoleic acid percentage observed in gilthead sea bream flesh (from 6% to 24%) being greater than in seabass muscle [32]. Similarly, when Menoyo et al. [33] replaced 60 or 80% of FO by SO in diets with 17.6% added fat, the increases in the linoleic acid content of gilthead sea bream (from 6% to 24% with the diet with 60% of SO and to 28% with the diet with 80% of SO) were higher than the one observed in our study for European seabass. Although this fact might be due to the different species studied, it can be also related to the greater differences in the linoleic acid content between their diets (5% in FO diet, 30% in the diet with 60% of SO, and 39% in the one with 80% of SO) than among the diets used in this study (Table 3).

Regarding MUFA, the highest MUFA values in the present study were found in fillets from fish on OPO and OPAO diets (≈48%), being higher than those found in FO and SO fillets. This agreed with the higher MUFA content in OPO and OPAO diets (Table 3) resulting from the addition of these fats of olive origin (Table 1). Likewise, the study carried out by Mourente et al. [34] showed an increment of oleic acid in fish flesh (from 16% to 28%) when European seabass was fed with a diet with 17.7% of added fat and a 60% of FO replacement by olive oil. However, a great number of factors beyond the fat source used in the diet can determine the FA profile of fish, such as the fish species used, the feeding period, the added fat percentage, or the proportion of FO replacement. For example, Nasopoulou et al. [35] did not obtain significant differences in oleic acid content for gilthead sea bream muscle probably because in diets, FO was substituted by only 8% of OPO. Another variable that affects the magnitude of FA modification is the type of muscle studied and its proportion between polar lipids, with structural functions, and neutral lipids [36, 37].

### 3.2. Tocopherol and Tocotrienol Composition of Fish Fillets

Vitamin E has an important role during fish rearing, as it helps preventing some diseases and contributes to a correct development of fish [38]. Moreover, the T and T3 deposited in fish fillets might have an important role in lipid oxidation, and thus, in their shelf life. The T and T3 contents in fresh and refrigerated European seabass fillets are presented in Table 5.

The contents of total T + T3 and *α*-T, which was the main tocol present in fish fillets, were not significantly affected by refrigeration. The refrigerated storage significantly reduced the *β*-T, *γ*-T, and *β*-T3 concentration in fillets coming from all diets. Literature results on the T and T3 stability during storage are controversial, as they might depend on the storage conditions and on the many factors that can affect T and T3 content in fish, such as the biology of the species, the source of vitamin E used in the diet, the presence of interacting compounds in the diet (such as selenium, vitamin C, or astaxanthin), or the PUFA content [39]. For example, mackerel-minced muscle kept into plastic bags significantly reduced its *α*-T content from 3.6 mg/kg to 1.0 mg/kg after 3 days of refrigeration at 4°C, and it continued dropping to 0.6 mg/kg after 7 and 11 days [40]. Another trial also showed that when rainbow trout specimens coming from a diet with 100 mg/kg of added *α*-tocopheryl acetate were packed into black nylon bags and refrigerated for 9 days at 1°C, the *α*-T concentrations in fillets decreased from 30.1 mg/kg to 19.1 mg/kg [41]. However, when seabass specimens fed diets with 4 different levels of *α*-tocopheryl acetate were kept in boxes, covered with flake-ice and black nylon bags, and stored in a refrigerated room at 1°C for 12 days, no significant variations in the vitamin E content (*α*-T + *γ*-T) of fillets were observed [42].

The total T + T3 content and the tocol profile of fresh and refrigerated fillets depended on the diet (Table 5), with the differences being in concordance with those observed between diets (Table 3) and between fat sources (Table 1). As animals are unable to synthetize T and T3, it is mandatory to have enough levels in diets to, at least, fulfill the essential requirements [43]. Since it might be difficult to reach them only with the T and T3 supplied by the added fat (Table 1) and by the rest of the feed ingredients, the addition of vitamin E in fish diets is a common practice in aquaculture. In this study, the vitamin-mineral premix supplied 192.23 mg of *α*-tocopheryl acetate per kg of feed (Table 2), making *α*-T the main tocol in all diets (Table 3) and, consequently, in fish fillets (Table 5). This also attenuated the differences in the tocol profile and in the total T + T3 content observed between the diets (Table 3). However, the influence of the type of fat used in each diet was still noticeable in the diets (Table 3) and thus reflected in the total T + T3 content and tocol profile of fresh and refrigerated fillets (Table 5). For instance, the partial substitution of FO by vegetable oils in fish diets increased T + T3 levels and modified the tocol profile of fish fillets (Table 5) because, among the fat sources, FO had the lowest total T + T3 concentration (Table 1). Regarding the vegetable fats, the total T + T3 levels were similar for OPO and OPAO, and the highest amount was found for SO, and even if SAO presented the second highest T + T3 amount, it was 7.8 times lower than that of S (Table 1). Consequently, SO fish fillets showed the highest T + T3 content and FO fish fillets the lowest ones (Table 5). Concerning the tocol profile of fish fillets, the highest *α*-T content was found for fillets from OPO diet, and similar *α*-T levels were found for OPAO and SO fillets, whereas fillets from SO diet had the highest levels of *γ*-T and *δ*-T, followed by the ones from SAO diet. This is also in concordance with the tocol profile of diets (Table 3) and fat sources (Table 1); as in fat sources, the highest *γ*-T levels were found for SO followed by SOA, with *γ*-T being the main compound in SO and *α*-T in SOA, which evidenced the presence of AO of sunflower origin [31]. The use of OPAO diet led to a similar T + T3 content and profile in fish fillets as OPO diet, except for a higher *β-*T content, which is in concordance with the similar tocol profile between these two fat sources (Table 1). Comparing SO with SAO, SO had a much higher *α*-T content than SAO (Table 1), but these differences were attenuated in the diets (Table 3) and led to fish fillets with similar *α*-T content (Table 5). Some modifications in the tocol profile of fish flesh when vegetable oils are added to fish diets have been reported before. For instance, Regost et al. [44] demonstrated that the *α*-T content was lower in Atlantic salmon fed with FO and SO diets than with low-erucic acid rapeseed oil, and that the highest *γ*-T level was observed for SO diet. Ng et al. [45] showed an increase in total T + T3 levels (from 7.5 to 19.3 mg/kg) and T3 content (from 1.0 mg/kg to more than 5.0 mg/kg) of catfish muscle with the replacement of FO by palm fatty acid distillate in the diet (from 0% to 100%). Also, Trullàs et al. [46] reported a greater content of *β* − T + *γ* − T and total T + T3 in rainbow trout when FO was replaced by 75% of different types of experimental rapeseed oils in diets with 20.1% added fat.

### 3.3. Lipid Hydroperoxide Content and Oxidative Stability of Fish Fillets

**Figure 1:**
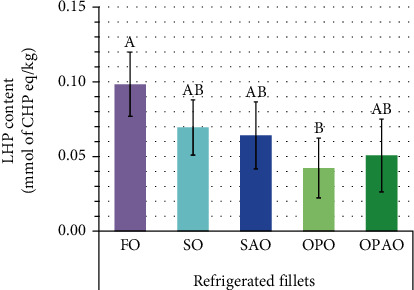
Primary oxidation of refrigerated fillets: lipid hydroperoxide content (obtained after an incubation time of 30 min) from the five dietary treatments of interest. The results were represented as mean ± standard deviation (*n* = 5) of CHP concentrations (mmol/kg). The differences between dietary treatments found in refrigerated fillets with Scheffé's post hoc (*n* = 25) were noted as A>B. Abbreviations: For diet abbreviations (FO, SO, SAO, OPO, and OPAO), see Table 2; LHP: lipid hydroperoxide; CHP: cumene hydroperoxide.

Primary oxidation of fresh and refrigerated fillets was evaluated by the LHP content obtained by the FOX method after a 30 min incubation. The LHP content in fresh fillets was too low to be quantifiable. However, the refrigerated storage increased the LHP content, with the levels being higher in refrigerated FO fillets (0.10 mmol/kg) than in OPO fillets (0.04 mmol/kg) (Figure 1). This might be related to the different FA unsaturation of fillets from different dietary treatments (Table 4), as FO fillets showed the highest content of unsaturated FA, where as OPO fillets had the lowest.

**Figure 2:**
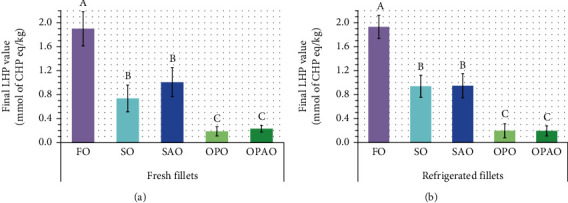
Oxidative stability: final lipid hydroperoxide value obtained after an incubation time of 96 h for fresh fillets (a) and refrigerated fillets (b) from the five diets of interest. The results were represented as mean ± standard deviation (*n* = 5) of CHP concentrations (mmol/kg). The differences between dietary treatments found in fresh or refrigerated fillets with Scheffé's post hoc test (*n* = 25) were noted as A>B>C. Abbreviations: For diet abbreviations (FO, SO, SAO, OPO, and OPAO), see Table 2; LHP: lipid hydroperoxide; CHP: cumene hydroperoxide.

The oxidative stability of the different samples (the higher the formation of LHP, the lower the oxidative stability) was evaluated by measuring the LHP by the FOX assay after an incubation period of 96 h (Final LHP value, Figure 2).

The effect of the interaction between the refrigeration time and the diet on the final LHP value was not significant. Also, the refrigerated storage for 6 days at the conditions assayed in this study did not affect the oxidative stability of the fillets. However, there were significant differences for this parameter between the diets (Figure 2) that were in concordance with the FA profile and the T and T3 content of fish fillets. The lowest oxidative stability was found for FO fillets, and it could be associated with its higher EPA and DHA contents, which are FA with a great tendency to suffer lipid oxidation [47]. This fact was supported by the significant Pearson's correlations between the final LHP value and EPA (*r* = 0.836; *p* < 0.001) and DHA percentages (*r* = 0.850; *p* < 0.001). As Figure 2 shows, SO and SAO diets led to fish fillets with an intermediate oxidative stability. Fillets coming from both diets were rich in linoleic and linolenic acid contents (Table 4), which are less susceptible to oxidize than EPA and DHA but more than MUFA. In addition, SO and SAO fillets had a significantly higher T + T3 content than FO fillets (Table 5), which might help preventing oxidation reactions. In fact, the final LHP value was negatively correlated with the content of the main tocol (*α*-T) in fish fillets (*r* = −0.807; *p* < 0.001). Despite SO fillets presented the highest T + T3 levels (Table 5), FA unsaturation was similar in SO and SAO fillets (Table 4), which led to similar behaviors in terms of oxidative stability. The highest oxidative stability was found for OPO and OPAO fillets, and this might also be related with their FA profile (Table 4), in which MUFA were the main type of FA, and PUFA had less relevance than in the other fillets. This is in agreement with the negative correlation found between final LHP value and oleic acid percentage (*r* = −0.820; *p* < 0.001). Therefore, lipid oxidative stability was clearly influenced by the botanical origin of the fat source used, but there was not an observable impact of the type (crude or acid) of vegetable oil. In this sense, the upcycling of SAO or OPAO as a replacement of FO in fish diets could improve the lipid oxidative stability in a similar way than their corresponding crude oils.

### 3.4. TBA Values of Fish Fillets

**Figure 3:**
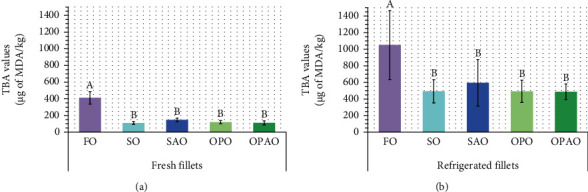
Secondary oxidation: TBA values of fresh fillets (a) and refrigerated fillets (b) coming from the different dietary treatments. The results were represented as mean ± standard deviation (*n* = 5) of TBA values (*μ*g of MDA/kg). The differences between dietary treatments found in fresh or refrigerated fillets with Scheffé's post hoc test (*n* = 25) were noted as A>B. Abbreviations: For diet abbreviations (FO, SO, SAO, OPO, and OPAO), see Table 2; MDA: malondialdehyde.

The presence of MDA as secondary oxidation product in fish fillets from the different diets was evaluated by the TBA values (Figure 3).

The TBA values of fish fillets were significantly affected by the refrigeration time (*p* < 0.001) and the diet used (for fresh fillets *p* < 0.001 and for refrigerated fillets *p* = 0.006), although there was no significant effect of the interaction between the two factors. The TBA values increased from the range of 112 *μ*g MDA/kg–412 *μ*g MDA/kg to 488 *μ*g MDA/kg–1051 *μ*g MDA/kg during the refrigerated storage of fish fillets packed in modified atmosphere. Generally, the results found in the literature are in concordance with the development of secondary oxidation reactions in fish during storage observed in our study. For instance, Kyrana and Lougovois [48] also found increases in the TBA values (from 370 to 650 *μ*g MDA/kg) in skinned fillets from ungutted European seabass stored in ice inside a refrigerator (0°C–4°C) for 22 days. Likewise, TBA values gradually increased in fillets from rainbow trout packed into black nylon bags and stored at 1°C for 9 days and revealed that a higher *α*-tocopheryl acetate content in the feed led to a higher *α*-T in trout fillets and a lower TBA values [41].

Concerning the effect of the diet on fillet TBA values, significant differences were observed between FO and the other dietary treatments (Figure 3), with the highest TBA values being found in FO fillets. This agreed with FO fillets showing the lowest oxidative stability (Figure 2), a fact that might be associated with their highest n-3 LC-PUFA content and the lowest T + T3 levels. Accordingly, TBA values had a strong positive correlation with various LC-PUFA, such as arachidonic acid (C20:4 n-6) (*r* = 0.943; *p* < 0.001), C20:3 n-3 (*r* = 0.879; *p* < 0.001), EPA (*r* = 0.936; *p* < 0.001), and DHA (*r* = 0.942; *p* < 0.001). Our observations about the impact of the diet on TBA values are in agreement with those obtained by Regost et al. [44] in Atlantic salmon, who also observed higher TBA levels when FO was added (at 29.8%) to the diet instead of SO. Contrarily, Trullàs et al. [46] found no significant differences in fillets of rainbow trout fed with a FO diet (20%) or those with vegetable fat sources (75% of FO replacement), but in that case, FO fillets showed the highest content in *α*-T.

### 3.5. Content of Volatile Compounds of Fish Fillets

The formation of volatile compounds can lead to a loss of sensorial attributes, because they are contributors to the aromatic profile of the fish. Nevertheless, the influence of volatile compounds on the odor depends not only on their concentration but also on their odor threshold. In the present study, a total of twelve compounds were identified in fresh and refrigerated European seabass fillets, including seven aldehydes (propanal, pentanal, hexanal, heptanal, (Z)-4-heptenal, octanal, and nonanal), three alcohols (1-penten-3-ol, (Z)-2-penten-1-ol, and 1-octen-3-ol), one ketone (2-octanone), and one furane (2-pentylfuran) (Table 6). Generally, in meat and fish products, aldehydes are considered crucial compounds, as they are characterized for possessing a lower odor threshold than alcohols and ketones [49].

There was not a significant effect of the interaction between the two main factors (refrigeration time and diet) on any volatile compound. The content of all the identified compounds suffered a noticeable increment after the refrigerated storage (Table 6).

Regarding the differences observed between dietary treatments, in fresh fillets, heptanal was the only aldehyde significantly affected by the diet (*p* = 0.024), but Scheffé's post hoc test was not able to separate the means of the different diets (Table 6). Heptanal might be associated with the oxidation of n-9 MUFA (such as oleic acid) or n-6 PUFA (such as linoleic or arachidonic acids) [49, 50]. For the aldehydes in refrigerated fillets, significant differences between dietary treatments were only found for propanal (Table 6), with the levels in FO fillets (6 *μ*g/kg) being higher than in OPAO fillets (2 *μ*g/kg). Propanal has been linked to the oxidation of n-3 PUFA [51], and, in this case, its relation with the development of lipid oxidation could be confirmed by its strong correlation with the TBA values (*r* = 0.751; *p* < 0.001). In our study, the main aldehyde observed in fish fillets was hexanal, which has been associated with n-6 PUFA oxidation, and it is commonly used as an indicator of lipid oxidation [49, 50]. However, in this work, hexanal was not significantly affected by the diet, contrary to what was observed for propanal. This is in agreement with some authors that have proposed propanal and heptanal as better indicators of rancidity in fish, whereas hexanal would usually be better correlated with meat flavor deterioration [52, 53]. The other aldehydes found in fish fillets could also be linked to oxidation reactions of some specific FA, octanal and nonanal might be associated to n-9 MUFA oxidation, pentanal might come from the oxidative degradation of n-6 PUFA, and (Z)-4-heptenal might be formed during n-3 PUFA oxidation [49], but results did not reveal any significant effect of the dietary treatment on any of them.

Similar to aldehydes, the presence of some alcohols, as the three identified in fish fillets in this study, might be related with lipid oxidation. For example, 1-octen-3-ol was the most important alcohol found in fish fillets, which is well-known because of its low odor threshold [49] and might be generated from n-6 PUFA oxidation [54]. In this study, the highest 1-octen-3-ol content was found in FO fillets (50 *μ*g/kg in fresh fillets and 126 *μ*g/kg in refrigerated fillets). This is in agreement with the highest arachidonic acid amount obtained for FO fillets even though they did not have the highest total n-6 PUFA content (Table 4). In addition, 1-octen-3-ol was the volatile compound that most correlated with arachidonic acid content (*r* = 0.785, *p* < 0.001). In refrigerated fish fillets, the diet also had a significant impact on the content of the other two alcohols identified: 1-penten-3-ol and 2-pentenol. Refrigerated FO fillets showed higher levels of 1-penten-3-ol than refrigerated SO and SAO fillets and higher content of (Z)-2-penten-1-ol than SAO fillets (Table 6). This alcohol has been associated with the oxidation of n-3 PUFA, such as linolenic acid, EPA, and DHA [50, 55]. The isomer (E)-2-pentenol has been identified by other authors in sardines, turbot, mussels, and clams [56], whereas (Z)-2-penten-1-ol has been proposed as a potential marker for salmon freshness [57] and for oxidation of krill oil during storage [58]. In fact, some authors have related the formation of this last alcohol with the oxidation of n-3 PUFA, concretely of EPA and DHA [50, 59].

The only ketone identified in this study was 2-octanone, which was a minor volatile compound in fresh (≈4 *μ*g/kg) and refrigerated fish fillets (≈6 *μ*g/kg). Some studies have linked the formation of this ketone to the oxidation of unsaturated FA, but any of them have specified the type of FA that can be involved [60–62]. However, Parlapani et al. [63] reported a bacterial origin for 2-octanone in a model substrate of seabream (made of sterile flesh fish juice agar) only when it was inoculated with different bacteria (*Pseudomonas* and *Carnobacterium/Lactobacillus*) and stored in modified atmosphere (CO_2_: 60%, O_2_: 10%, and N_2_: 30%) for 18 days at 0°C or for 2 days at 15°C.

The last compound found in fish fillets was 2-pentylfuran which has been associated with n-6 PUFA oxidation [49]. The diet had a significant effect on the content of this compound in fresh fillets but not in refrigerated fillets. Fresh SAO led to higher values (22 *μ*g/kg) than FO, OPO, or OPAO fillets (10 *μ*g/kg–11 *μ*g/kg).

Despite some volatile compounds can be formed by other reactions not related to FA, such as amino acid degradation or microbial action [49], the relation between the volatile compound formation and lipid oxidation in this study was clear. The significant increase of all volatile compounds in fish fillets after the refrigeration agreed with the development of oxidation reactions (Figure 3). Moreover, strong correlations were found not only for different volatile compounds and the TBA values but also for the total aldehyde content (*r* = 0.715; *p* < 0.001). Also, the differences in volatile compounds observed between FO fillets and fillets from the rest of the diets agreed with their higher TBA values.

### 3.6. Color of Fish Fillets

The behavior of the color parameters (L^∗^, a^∗^, and b^∗^) in the different diets was the same in fresh than in refrigerated fillets (Table 7). The refrigerated storage had a significant impact on L^∗^ and a^∗^, whereas no effect was found for b^∗^. The lightness of the fillets increased after the refrigerated period and the a^∗^ values decreased, implying that refrigerated fillets lost redness and gained greenness compared with fresh fillets.

The Δ*E*_*R*_ parameter that compares instrumental color parameters between fresh and refrigerated fillets reflected that the greatest color stability was found for SO fillets (0.6), whereas FO fillets were the ones which experienced the highest color change after the refrigerated storage (2.4). Since Δ*E*_*R*_ values were in all cases lower than 5 [26], these differences in color parameters might not be perceived by the human eye.

Color differences among fresh fillets from the studied dietary treatments were significant for L^∗^ and a^∗^. The highest L^∗^ value was observed for SO fresh fillets, where as FO and OPAO fresh fillets had the lowest values. However, FO fresh fillets were the ones with highest a^∗^. Accordingly, compared to FO fresh fillets, the highest similarities in color were found for OPAO fresh fillets (Δ*E*_*D*_ = 0.8) and the greatest differences for SO fresh fillets (Δ*E*_*D*_ = 2.4). In the case of refrigerated fillets, significant differences were found only in a^∗^ between FO fillets and SO and OPO fillets. However, considering all three parameters, OPO fillets (Δ*E*_*D*_ = 0.8) had the greatest color similarities to FO refrigerated fillets, whereas OPAO fillets (Δ*E*_*D*_ = 0.9) were the least similar to FO fillets. As Δ*E*_*D*_ in all cases was lower than 5 [26], the color changes of fresh and refrigerated fillets due to the partial substitution of FO for SO, SAO, OPO, or OPAO might not be perceptible by the human eye.

The facts that are behind the color changes in fish and fish products are not as clear as in the case of meat [64] and are less studied in white fish muscle than in flesh rich in pigments, such as salmon. Two different trials showed that 60% or 80% FO replacement by vegetable oils (SO or linseed oil) reduced a^∗^ and b^∗^ in seabream fillets [30, 33]. In the present study, yellowness (b^∗^) was the most stable parameter as it was not influenced by the refrigeration, and there was no strong correlation between the lightness, redness, or yellowness and lipid oxidation parameters. Contrarily, another study related the increase of b^∗^ in dark muscle of yellowtail after 2 days of storage in ice to the development of lipid oxidation [65]. These differences between our results and the ones found in the literature can be due to the different conditions (type of muscles, fish species, or processing and storage conditions) used in each case.

### 3.7. Sensory Acceptance of Fish Fillets

Refrigeration had no significant effect on the overall acceptance of European seabass filets (Table 7). Despite ANOVA showed significant differences between dietary treatments in fresh fillets, Scheffé's post hoc test could not separate the means of the different diets. After the refrigerated storage, the diet used did not significantly affect the overall acceptance of fish fillets.

In our study, the significant increase of TBA values and volatile compound content observed during the refrigerated storage did not result in a reduction of the overall acceptability. A great number of trials associate the loss of the sensory acceptance with the secondary oxidation products measured by TBA values [66–68]. In seabass fillets, sensory rejection was found by trained panelists at TBA values between 700–2,400 *μ*g MDA/kg [67]. The TBA values found for fresh and refrigerated European seabass fillets in our study were not higher than these levels. This indicates that lipid oxidation during the processing and storage of fish fillets may not have developed enough to affect the sensorial properties, which agreed with the results obtained in the overall acceptance test.

In the literature, it has been reported that the use of vegetable oils in fish feeds can alter the sensory characteristics of fresh fish flesh [6]. However, in agreement with our observations, the results obtained by Montero et al. [69] revealed that the organoleptic properties of fresh European seabass fillets were not modified due to the inclusion of vegetable oils (60% of SO, rapeseed, or linseed oils) in diets. Also in other studies, the acceptability of fresh seabream fillets [30, 32] and of seabass fillets [32] was not affected by the dietary supplementation with vegetable oils (SO, rapeseed, or linseed oils at 60% or 80%).

## 4. Conclusions

The partial substitution of FO with SO, SAO, OPO, or OPAO in fish diets reduced EPA and DHA contents and increased T + T3 in fish fillets. Even though, the recommended EPA plus DHA amount for human daily intake (250 mg) could be covered with 100 g of fish fillets coming from all experimental diets. These changes in fillet composition led to a decrease of lipid oxidation reactions in fish fillets coming from SO, SAO, OPO, and OPAO diets, revealed by a lower final LHP value, a lower TBA value, and a lower formation of some volatile compounds (e.g., 1-octen-3-ol) in fresh and refrigerated fillets. Moreover, the partial substitution of FO for SO, SAO, OPO, or OPAO did not affect the overall acceptability and may not have influenced consumer's perception of the color of the fish fillets. According to the results obtained in this study, in general, the partial replacement of FO by AO instead of crude oils of similar FA composition similarly affected the oxidative parameters, the color, or the overall acceptance of fish fillets. Overall, the findings show that the final FA composition, T content, and oxidative stability of fish fillets might be more dependent on the botanical origin of acid oils (and thus, on their FA composition and T content), than on the fact that they are acid or crude oils, at least when their quality (MIU value) is close to the recommended values [11, 27].

A clear development of lipid oxidation during the refrigerated storage of European seabass fillets was observed with the increment of TBA values and of the volatile compound content of fish fillets, which occurred in all experimental diets. However, the storage conditions (CO_2_/N_2_/O_2_; 40/30/30; 2°C; 6 days) were adequate to prevent a loss of overall acceptance of fish fillets and might have prevented color changes perceptible by the human eye. Overall, from the quality flesh point of view, it could be possible to upcycle these refining by-products to partially substitute FO in fish diets instead of using crude oils of similar botanical origin.

## Figures and Tables

**Table 1:** Composition of the experimental fats used in this study.

	FO	SO	SAO	OPO	OPAO
MIU (g/100 g)^1^					
Moisture^2^	0.24 ± 0.01	0.05 ± 0.01	0.40 ± 0.01	0.36 ± 0.01	0.31 ± 0.01
Impurities	0.30 ± 0.05	0.21 ± 0.03	0.89 ± 0.15	0.44 ± 0.12	1.94 ± 0.21
Unsaponifiable	2.01 ± 0.12	0.53 ± 0.25	2.35 ± 0.34	1.64 ± 0.35	3.90 ± 0.35
Total	2.55 ± 0.13	0.80 ± 0.25	3.64 ± 0.37	2.44 ± 0.37	6.15 ± 0.41

FA composition (%)^1; 3^					
C14:0	3.7 ± 0.13	ND	ND	ND	ND
C16:0	21.8 ± 0.50	10.7 ± 0.04	11.2 ± 0.05	13.3 ± 2.18	11.5 ± 0.05
C18:0	6.4 ± 0.09	3.4 ± 0.11	3.5 ± 0.01	2.7 ± 0.06	3.1 ± 0.01
SFA	34.9 ± 0.53	15.0 ± 0.24	16.3 ± 0.06	16.8 ± 2.18	16.0 ± 0.05
C16:1 n-7	4.7 ± 0.35	0.1 ± 0.01	0.1 ± 0.01	0.7 ± 0.02	1.1 ± 0.02
C18:1 n-9	16.0 ± 0.20	23.5 ± 0.02	30.5 ± 0.08	68.7 ± 1.76	63.0 ± 0.19
C18:1 n-7	2.8 ± 0.03	1.7 ± 0.02	1.4 ± 0.01	2.0 ± 0.12	2.1 ± 0.06
MUFA	28.4 ± 0.41	25.6 ± 0.06	32.2 ± 0.08	71.7 ± 1.77	66.5 ± 0.20
C18:2 n-6	1.9 ± 0.02	53.1 ± 0.35	48.3 ± 0.03	10.4 ± 0.15	16.5 ± 0.11
n-6 PUFA	2.8 ± 0.04	53.2 ± 0.35	48.3 ± 0.03	10.5 ± 0.15	16.6 ± 0.12
C18:3 n-3	0.9 ± 0.01	6.1 ± 0.01	3.0 ± 0.01	0.9 ± 0.01	0.7 ± 0.01
C20:5 n-3	6.3 ± 0.92	ND	ND	ND	ND
C22:6 n-3	26.0 ± 0.15	ND	ND	ND	ND
n-3 PUFA	33.6 ± 1.04	6.1 ± 0.01	3.0 ± 0.01	0.9 ± 0.01	0.7 ± 0.01
Total PUFA	36.4 ± 1.04	59.2 ± 0.35	51.3 ± 0.04	11.3 ± 0.15	17.3 ± 0.12
*Trans* C18:1	0.3 ± 0.01	0.2 ± 0.08	0.1 ± 0.02	0.1 ± 0.04	0.3 ± 0.2

T and T3 content (mg/kg)^1^					
*α*-T	41.18 ± 1.51	170.93 ± 4.71	40.83 ± 0.40	66.44 ± 0.57	65.61 ± 2.24
*β*-T	0.32 ± 0.03	20.04 ± 0.03	2.90 ± 0.03	0.47 ± 0.15	1.51 ± 0.10
*γ*-T	0.21 ± 0.05	535.93 ± 10.96	36.44 ± 0.61	2.22 ± 0.01	1.91 ± 0.09
*δ*-T	0.65 ± 0.32	53.63 ± 1.63	20.95 ± 0.84	ND	0.55 ± 0.01
*β*-T3	0.41 ± 0.17	4.35 ± 0.62	0.36 ± 0.03	3.11 ± 0.05	1.29 ± 0.09
*γ*-T3	ND	9.23 ± 0.28	ND	ND	0.99 ± 0.05
Total (T + T3)	42.77 ± 1.92	794.11 ± 13.72	101.48 ± 1.92	72.64 ± 0.44	71.86 ± 2.46

Lipid class composition (%)^1; 4^					
TAG	85.7 ± 0.01	93.9 ± 0.08	29.3 ± 0.30	77.5 ± 0.15	36.3 ± 0.10
DAG	6.9 ± 0.03	4.2 ± 0.08	16.1 ± 0.24	8.4 ± 0.15	17.4 ± 0.01
MAG	4.4 ± 0.03	0.5 ± 0.01	1.3 ± 0.28	0.9 ± 0.17	1.4 ± 0.10
FFA	3.1 ± 0.06	1.5 ± 0.01	53.3 ± 0.34	13.2 ± 0.12	45.0 ± 0.01

Acid value (mg KOH/g)^1^	14.3 ± 0.19	2.8 ± 0.11	116.6 ± 1.8	23.3 ± 0.11	96.8 ± 0.45

Peroxide value (meq O_2_/kg)^1^	2.7 ± 0.44	1.5 ± 0.12	1.7 ± 0.08	6.0 ± 0.72	1.7 ± 0.46

Abbreviations: FO: fish oil; SO: crude soybean oil; SAO: soybean-sunflower acid oil; OPO: crude olive pomace oil; OPAO: olive pomace acid oil; MIU: moisture and volatile matter + insoluble impurities + unsaponifiable matter; FA: fatty acid; SFA: saturated fatty acids (sum of C14:0, C15:0, C16:0, C17:0, C18:0, C20:0, C22:0, and C24:0); MUFA: monounsaturated fatty acids (sum of C16:1 n-9, C16:1 n-7, C17:1 n-7, C18:1 n-9, C18:1 n-7, C20:1 n-9, C22:1 n-9, and C24:1 n-9); PUFA: polyunsaturated fatty acids (n-6 PUFA: sum of C18:2 n-6, C18:3 n-6, C20:2 n-6, C20:3 n-6, and C20:4 n-6; n-3 PUFA: sum of C18:3 n-3, C20:3 n-3, C20:5 n-3, and C22:6 n-3; total PUFA: sum of n-6 PUFA and n-3 PUFA); T: tocopherol; T3: tocotrienol; TAG: triacylglycerols; DAG: diacylglycerols; MAG: monoacylglycerols; FFA: free fatty acids; ND: not detected. ^1^Mean ± standard deviation of the three determinations. ^2^ It included moisture and other compounds that volatilize under the determination conditions. ^3^ The percentage of each FA was obtained by peak area normalization. ^4^ The percentage of each lipid class was obtained by peak area normalization.

**Table 2:** Ingredients, proximate composition, and gross energy of the five diets used in this study.

	FO	SO	SAO	OPO	OPAO
*Ingredient composition (g/kg)*					
Wheat meal	110.3	110.3	110.3	110.3	110.3
Wheat gluten	155.9	155.9	155.9	155.9	155.9
Soya protein concentrate	266.0	266.0	266.0	266.0	266.0
Fish meal	202.4	202.4	202.4	202.4	202.4
Hydrolyzed fish protein	25.3	25.3	25.3	25.3	25.3
Krill meal	25.5	25.5	25.5	25.5	25.5
Soybean lecithin	9.6	9.6	9.6	9.6	9.6
Fish oil	153.9	38.5	38.5	38.5	38.5
Experimental fat	0.0	115.4	115.4	115.4	115.4
L-lysine	2.9	2.9	2.9	2.9	2.9
DL-methionine	1.0	1.0	1.0	1.0	1.0
Choline chloride	4.8	4.8	4.8	4.8	4.8
Betaine	1.0	1.0	1.0	1.0	1.0
Vitamin and mineral premix^1^	19.2	19.2	19.2	19.2	19.2
Vitamin C	1.0	1.0	1.0	1.0	1.0
Guar gum	19.2	19.2	19.2	19.2	19.2
*Proximate composition (g/kg)*					
Dry matter	926.1	925.7	932.3	926.2	930.4
Crude protein	418.3	405.3	396.2	413.1	414.3
Ether extract	190.5	190.4	182.8	186.9	180.0
Ash	72.2	72.4	73.2	72.4	73.4
Gross energy (MJ/kg)	21.7	21.8	21.8	21.7	22.0

Abbreviations: FO: fish oil diet; SO: crude soybean oil diet; SAO: soybean-sunflower acid oil diet; OPO: crude olive pomace oil diet; OPAO: olive pomace acid oil diet. ^1^ Provides per kg of feed: vitamin A (38,460 IU), vitamin D3 (3,8460 UI), vitamin E (192.23 mg of *α*-tocopheryl acetate), vitamin K3 (48.08 mg), vitamin B1 (57.69 mg), vitamin B2 (57.69 mg), calcium pantothenate (192.23 mg), nicotinic acid (384.60 mg), vitamin B6 (38.46 mg), vitamin B9 (28.84 mg), vitamin B12 (0.19 mg), biotin (5.77 mg), inositol (961.50 mg), betaine (961.50 mg), cobalt carbonate (1.25 mg), cupric sulphate (17.31 mg), ferrous sulphate (11.54 mg), potassium iodide (0.96 mg), manganese oxide (18.46 mg), sodium selenite (0.02 mg), zinc sulphate (14.42 mg), calcium carbonate (3,577 mg), potassium chloride (463.44 mg), and sodium chloride (769.20 mg).

**Table 3:** Fatty acid, tocopherol and tocotrienol, and lipid class compositions of the experimental diets^1^.

	FO^2^	SO^2^	SAO^2^	OPO^2^	OPAO^2^
*FA composition (%)* ^3^					
C14:0	3.74 ± 0.05	1.40 ± 0.01	1.52 ± 0.06	1.44 ± 0.07	1.51 ± 0.05
C16:0	23.43 ± 1.73	15.13 ± 0.15	15.79 ± 0.59	15.77 ± 0.75	15.29 ± 0.06
C18:0	6.77 ± 0.56	4.45 ± 0.10	3.88 ± 0.85	3.50 ± 0.58	4.16 ± 0.04
SFA	35.16 ± 1.82	21.69 ± 0.18	22.18 ± 1.04	21.27 ± 0.95	21.69 ± 0.10
C16:1 n-7	4.39 ± 0.18	1.54 ± 0.01	1.63 ± 0.06	1.98 ± 0.09	2.20 ± 0.01
C18:1 n-9	16.28 ± 0.61	22.20 ± 0.58	27.20 ± 1.31	50.45 ± 1.56	46.12 ± 0.58
C18:1 n-7	2.71 ± 0.07	1.87 ± 0.04	1.55 ± 0.10	1.58 ± 0.13	2.10 ± 0.45
C20:1 n-9	1.52 ± 0.04	0.67 ± 0.01	0.66 ± 0.02	0.76 ± 0.03	0.71 ± 0.01
MUFA	25.09 ± 0.64	26.38 ± 0.58	31.15 ± 1.32	54.98 ± 1.57	51.32 ± 0.73
C18:2 n-6	6.16 ± 0.18	37.03 ± 0.78	33.13 ± 1.46	11.73 ± 0.57	15.03 ± 0.01
C20:4 n-6	2.10 ± 0.09	0.64 ± 0.01	0.67 ± 0.02	0.68 ± 0.03	0.68 ± 0.01
n-6 PUFA	9.04 ± 0.20	38.21 ± 0.78	34.50 ± 1.47	12.99 ± 0.57	16.31 ± 0.04
C18:3 n-3	1.21 ± 0.02	4.72 ± 0.01	2.60 ± 0.08	1.21 ± 0.06	1.05 ± 0.06
C20:5 n-3	6.72 ± 0.31	2.20 ± 0.01	2.44 ± 0.07	2.36 ± 0.12	2.45 ± 0.01
C22:6 n-3	21.98 ± 0.89	6.51 ± 0.01	6.84 ± 0.22	6.88 ± 0.41	6.92 ± 0.07
n-3 PUFA	30.70 ± 0.94	13.72 ± 0.02	12.17 ± 0.24	10.76 ± 0.43	10.69 ± 0.10
Total PUFA	39.74 ± 0.97	51.93 ± 0.78	46.67 ± 1.48	23.75 ± 0.72	26.99 ± 0.11

*T and T3 profile (mg/kg)*					
*α*-T	168.99 ± 5.97	230.03 ± 3.49	186.32 ± 3.90	235.93 ± 4.29	217.13 ± 5.57
*β*-T	0.95 ± 0.06	3.61 ± 0.22	3.43 ± 0.10	1.93 ± 0.15	3.19 ± 0.18
*γ*-T	1.40 ± 0.02	69.95 ± 17.29	27.37 ± 0.66	4.11 ± 0.18	3.67 ± 0.38
*δ*-T	ND	21.30 ± 4.50	16.68 ± 0.28	ND	ND
Total (T+T3)	177.07 ± 6.23	329.20 ± 29.37	240.30 ± 4.27	253.03 ± 4.79	233.95 ± 5.15

*Lipid class composition (%)* ^4^					
TAG	81.56 ± 0.77	86.74 ± 0.03	47.35 ± 0.10	76.06 ± 0.07	50.90 ± 0.26
DAG	8.76 ± 0.72	6.35 ± 0.33	14.17 ± 0.05	10.04 ± 0.01	15.53 ± 1.03
MAG	0.16 ± 0.02	0.47 ± 0.08	2.39 ± 0.01	0.93 ± 0.07	2.61 ± 0.21
FFA	9.53 ± 0.03	6.43 ± 0.22	36.09 ± 0.16	12.97 ± 0.14	30.97 ± 1.50

Abbreviations: For diet abbreviations (FO, SO, SAO, OPO, and OPAO), see Table 2; FA: fatty acid; SFA: saturated fatty acids (sum of C14:0, C15:0, C:16:0; C18:0, and C22:0); MUFA: monounsaturated fatty acids (sum of C16:1 n-9, C16:1 n-7, C18:1 n-9, C18:1 n-7, and C20:1 n-9); PUFA: polyunsaturated fatty acids (n-6 PUFA: sum of C18:2 n-6, C18:3 n-6, C20:2 n-6, and C20:4 n-6; n-3 PUFA: sum of C18:3 n-3, C20:3 n-3, C20:5 n-3, and C22:6 n-3; total PUFA: sum of n-6 PUFA and n-3 PUFA); T: tocopherol; T3: tocotrienol; TAG: triacylglycerols; DAG: diacylglycerols; MAG: monoacylglycerols; FFA: free fatty acids; ND: not detected. ^1^ The analytical methods applied are described in supplementary material. ^2^ Mean ± standard deviation of the two determinations. ^3^ The percentage of each FA was obtained by peak area normalization. ^4^ The percentage of each lipid class was obtained by peak area normalization.

**Table 4:** Fatty acid profile of fresh fillets coming from fish fed with the five experimental diets.

FA (%)	FO^1^	SO^1^	SAO^1^	OPO^1^	OPAO^1^	SEM^2^	*p* ^3^
C14:0	2.5^a^	1.5^c^	1.7^b^	1.6^bc^	1.6^bc^	0.026	**<0.001**
C16:0	19.2^a^	16.4^bc^	16.4^bc^	16.6^b^	16.0^c^	0.130	**<0.001**
C18:0	4.7^a^	4.6^a^	4.3^b^	4.0^c^	3.9^c^	0.053	**<0.001**
SFA	27.5^a^	23.2^b^	23.2^b^	22.9^bc^	22.3^c^	0.187	**<0.001**
C16:1 n-7	4.0^a^	2.3^c^	2.5^bc^	2.6^b^	2.7^b^	0.053	**<0.001**
C18:1 n-9	25.8^d^	27.8^d^	30.6^c^	43.3^a^	40.6^b^	0.538	**<0.001**
C18:1 n-7	1.8^a^	1.6^ab^	1.4^ab^	1.2^b^	1.6^ab^	0.118	**0.022**
C20:1 n-9	1.9^a^	1.4^d^	1.5^cd^	1.6^b^	1.6^bc^	0.027	**<0.001**
MUFA	34.0^bc^	33.4^c^	36.5^b^	49.2^a^	47.0^a^	0.560	**<0.001**
C18:2 n-6	11.4^e^	26.2^a^	23.7^b^	12.9^d^	15.1^c^	0.310	**<0.001**
C20:4 n-6	1.7^a^	0.7^b^	0.8^b^	0.8^b^	0.8^b^	0.024	**<0.001**
n-6 PUFA	13.9^d^	28.3^a^	25.6^b^	14.4^d^	16.6^c^	0.314	**<0.001**
C18:3 n-3	2.0^c^	3.8^a^	2.6^b^	1.9^d^	1.9^cd^	0.028	**<0.001**
C20:5 n-3	5.3^a^	2.7^c^	3.0^b^	2.9^bc^	3.0^b^	0.055	**<0.001**
C22:6 n-3	16.1^a^	7.7^b^	8.2^b^	7.9^b^	8.1^b^	0.293	**<0.001**
n-3 PUFA	24.3^a^	14.8^b^	14.4^b^	13.2^b^	13.7^b^	0.331	**<0.001**
Total PUFA	38.2^b^	43.0^a^	40.0^b^	27.6^d^	30.3^c^	0.483	**<0.001**

Abbreviations: For diet abbreviations (FO, SO, SAO, OPO, and OPAO), see Table 2; FA: fatty acid; SFA: saturated fatty acids (sum of C14:0, C16:0, C17:0, C18:0, C20:0, and C22:0); MUFA: monounsaturated fatty acids (sum of C16:1 n-9, C16:1 n-7, C18:1 n-9, C18:1 n-7, and C20:1 n-9); PUFA: polyunsaturated fatty acids (n-6 PUFA: sum of C18:2 n-6, C18:3 n-6, C20:2 n-6, and C20:4 n-6; n-3 PUFA: sum of C18:3 n-3, C20:3 n-3, C20:5 n-3, and C22:6 n-3; total PUFA: sum of n-3 PUFA and n-6 PUFA). See Table S1 in supplementary material for the complete FA composition including minor FA results. ^1^ Data were expressed as the mean of the five replicates from each dietary treatment (*n* = 5). ^2^ Standard error of the mean. ^3^*p* values obtained by ANOVA (*n* = 25). Values in bold were significant (*p* < 0.05). Differences between dietary treatments found with Scheffé's post hoc test were noted in the same row as *a* > *b* > *c* > *d* > *e*.

**Table 5:** Tocopherol and tocotrienol content of fresh and refrigerated fish fillets coming from fish fed with the five experimental diets.

	*α*-T (mg/kg)	*β*-T (mg/kg)	*γ*-T (mg/kg)	*δ*-T (mg/kg)	*β*-T3 (mg/kg)	T + T3 (mg/kg)
**Effect of the diet on fresh fillets**						
FO^1^	6.98^c^	0.09^c^	0.43^c^	0.05^c^	0.12^b^	7.68^c^
SO^1^	9.12^ab^	0.29^a^	5.79^a^	1.08^a^	0.16^b^	16.43^a^
SAO^1^	8.02^bc^	0.22^b^	1.96^b^	0.80^b^	0.16^b^	11.16^b^
OPO^1^	10.44^a^	0.19^b^	0.71^c^	0.04^c^	0.22^a^	11.60^b^
OPAO^1^	9.17^ab^	0.26^a^	0.72^c^	0.06^c^	0.23^a^	10.44^b^
SEM^2^	0.351	0.008	0.124	0.020	0.009	0.457
*p_diet_* ^3^	**< 0.001**	**< 0.001**	**< 0.001**	**< 0.001**	**< 0.001**	**< 0.001**
**Effect of the diet on refrigerated fillets**						
FO^1^	6.48^c^	0.08^d^	0.39^c^	0.04^c^	0.08^b^	7.06^c^
SO^1^	8.75^ab^	0.26^a^	5.20^a^	0.97^a^	0.12^ab^	15.28^a^
SAO^1^	8.15^b^	0.21^b^	1.84^b^	0.73^b^	0.13^ab^	11.05^b^
OPO^1^	10.03^a^	0.17^c^	0.64^c^	0.04^c^	0.19^a^	11.07^b^
OPAO^1^	9.38^ab^	0.23^ab^	0.63^c^	0.04^c^	0.18^a^	10.45^b^
SEM^2^	0.287	0.008	0.127	0.016	0.018	0.395
*p_diet_* ^3^	**< 0.001**	**< 0.001**	**< 0.001**	**< 0.001**	**< 0.001**	**< 0.001**
**Effect of the refrigeration**						
Fresh Fillets^4^	8.74	0.21	1.92	0.41	0.18	11.46
Refrigerated Fillets^4^	8.56	0.19	1.74	0.35	0.14	10.98
SEM^2^	0.144	0.004	0.056	0.008	0.006	0.191
*p_refrigeration_* ^5^	0.363	**< 0.001**	**0.028**	**< 0.001**	**< 0.001**	0.089
**Effect of the interaction between refrigeration and diet**						
*p_refrigeration x diet_* ^5^	0.724	0.617	0.173	**0.011**	0.891	0.686

Abbreviations: For diet abbreviations (FO, SO, SAO, OPO and OPAO) see Table 2; T, tocopherol; T3, tocotrienol. ^1^ Mean of the different replicates for each dietary treatment (n = 5). ^2^ Standard error of the mean. ^3^*p* values obtained from ANOVA (n = 25) of fresh or refrigerated fillets. Values in bold were significant (*p* < 0.05). The differences between dietary treatments found in fresh or refrigerated fillets with Scheffé's post hoc test (n = 25) were noted in the same column as a > b > c > d. ^4^ Pooled means of fresh or refrigerated fillets coming from the five dietary treatments (n = 25). ^5^*p* values obtained for the refrigeration (*p_refrigeration_*) and the interaction between the refrigeration time and the diet (*p_refrigeration x diet_*) from multifactor ANOVA (n = 50). Values in bold were significant (*p* < 0.05).

**Table 6:** Volatile compounds (*μ*g/kg) of fresh and refrigerated European seabass fillets.

	Propanal	Pentanal	Hexanal	Heptanal	(Z)-4-Heptenal	Octanal	Nonanal	1-Penten-3-ol	(Z)-2-Penten-1-ol	1-Octen-3-ol	2-Octanone	2-Pentylfuran
*Effect of the diet on fresh fillets*												
FO^1^	2.7	7.0	46.6	9.6	1.2	3.1	7.0	34.8	5.7	49.8^a^	4.5	9.9^b^
SO^1^	1.7	5.4	42.8	5.4	0.7	1.9	6.4	23.2	4.6	24.3^b^	3.0	13.0^ab^
SAO^1^	1.7	5.1	36.9	4.8	1.2	2.2	6.2	15.9	2.2	21.8^b^	3.5	22.5^a^
OPO^1^	2.2	6.7	32.3	4.6	0.8	2.1	5.8	26.8	5.9	23.9^b^	3.8	9.5^b^
OPAO^1^	1.9	4.1	25.6	4.4	0.8	1.3	4.7	22.6	5.0	21.2^b^	4.0	11.0^b^
SEM^2^	0.458	1.616	9.024	1.166	0.319	0.500	1.157	4.447	1.141	4.065	0.465	2.296
*p_diet_* ^3^	0.516	0.708	0.511	**0.024**	0.765	0.192	0.722	0.083	0.202	**< 0.001**	0.200	**0.004**

*Effect of the diet on refrigerated fillets*												
FO^1^	6.0^a^	14.3	100.6	15.1	2.1	4.6	11.8	84.9^a^	15.4^a^	126.5^a^	4.9	14.1
SO^1^	3.8^ab^	17.5	100.3	15.6	2.5	3.6	11.0	40.5^b^	7.9^ab^	64.6^b^	7.0	33.1
SAO^1^	3.6^ab^	9.0	91.2	8.2	2.3	3.7	9.0	42.6^b^	6.0^b^	66.9^b^	5.0	33.9
OPO^1^	3.3^ab^	14.8	62.0	9.8	1.7	3.2	10.1	49.9^ab^	10.7^ab^	60.4^b^	5.3	12.2
OPAO^1^	2.5^b^	8.9	57.2	13.9	2.5	4.6	10.0	46.4^ab^	8.8^ab^	65.1^b^	5.4	18.5
SEM^2^	0.560	2.824	12.710	3.301	0.910	0.909	3.010	7.829	1.586	11.956	0.519	6.236
*p_diet_* ^3^	**0.005**	0.192	0.060	0.469	0.971	0.774	0.977	**0.005**	**0.008**	**0.004**	0.067	0.068

*Effect of the refrigeration*												
Fresh Fillets^4^	2.1	5.6	36.8	5.8	0.9	2.1	6.0	24.6	4.7	28.2	3.8	13.2
Refrigerated Fillets^4^	3.8	12.9	82.2	12.5	2.2	3.9	10.4	52.9	9.8	76.7	5.5	22.4
SEM^2^	0.228	1.022	4.908	1.096	0.302	0.326	1.010	2.829	0.615	3.953	0.220	2.081
*p_refrigeration_* ^5^	**< 0.001**	**< 0.001**	**< 0.001**	**< 0.001**	**0.006**	**< 0.001**	**0.005**	**< 0.001**	**< 0.001**	**< 0.001**	**< 0.001**	**0.004**

*Effect of the interaction between refrigeration and diet*												
*p_refrigeration x diet_* ^5^	0.108	0.437	0.559	0.595	0.941	0.622	0.989	0.112	0.141	0.179	0.050	0.364

Abbreviations: For diet abbreviations (FO, SO, SAO, OPO and OPAO) see Table 2. ^1^ Mean of the different replicates for each dietary treatment (n = 5) expressed as 4-metil-2-pentanol equivalents. ^2^ Standard error of the mean. ^3^*p* values obtained from ANOVA (n = 25) of fresh or refrigerated fillets. Values in bold were significant (*p* < 0.05). The differences between dietary treatments found in fresh or refrigerated fillets with Scheffé's post hoc test were noted in the same column as a > b. ^4^ Pooled means of fresh or refrigerated fillets coming from the five dietary treatments (n = 25). ^5^*p* values obtained for the refrigeration (*p_refrigeration_*) and the interaction between the refrigeration time and the diet (*p_refrigeration x diet_*) from multifactor ANOVA (n = 50). Values in bold were significant (*p* < 0.05).

**Table 7:** Color parametres (L^∗^, a^∗^, b^∗^, and Δ*E* values) and consumer overall acceptance of fresh and refrigerated fish fillets.

	Color parameters					Overall acceptance
	L^∗^	a^∗^	b^∗^	*Δ*E_R_^1^ (Fresh vs refrigerated)	*Δ*E_D_^1^ (F vs other dietary treatments)	Sensory scores
*Effect of the diet on fresh fillets*						
FO^2^	73.33^b^	3.81^a^	9.11	2.35		6.57
SO^2^	75.35^a^	2.57^b^	8.99	0.64	2.38	5.97
SAO^2^	74.67^ab^	2.92^b^	8.87	1.39	1.63	5.67
OPO^2^	74.84^ab^	3.00^b^	9.35	1.18	1.73	5.30
OPAO^2^	73.24^b^	3.01^b^	8.94	1.65	0.82	5.03
SEM^3^	0.412	0.142	0.102			0.350
*p_diet_* ^4^	**0.004**	**< 0.001**	**0.032**			**0.023**

*Effect of the diet on refrigerated fillets*						
FO^2^	75.47	2.85^a^	9.32			6.13
SO^2^	75.61	2.04^b^	9.25		0.83	6.57
SAO^2^	75.70	2.28^ab^	8.88		0.89	5.77
OPO^2^	75.57	2.10^b^	9.51		0.78	5.40
OPAO^2^	74.73	2.33^ab^	9.09		0.93	5.70
SEM^3^	0.463	0.142	0.180			0.326
*p_diet_* ^4^	0.609	**0.006**	0.125			0.115

*Effect of the refrigeration*						
Fresh Fillets^5^	74.29	3.06	9.05			5.71
Refrigerated Fillets^5^	75.42	2.32	9.20			5.91
SEM^3^	0.196	0.064	0.065			0.151
*p_refrigeration_* ^6^	**< 0.001**	**< 0.001**	0.145			0.335

*Effect of the interaction between refrigeration and diet*						
*p_refrigeration x diet_* ^6^	0.266	0.508	0.806			0.481

Abbreviations: For diet abbreviations (FO, SO, SAO, OPO and OPAO) see Table 2; L^∗^, Lightness; a^∗^, redness; b^∗^ yellowness. ^1^*Δ*E values were calculated by Equation (1) (*Δ*E > 5 would mark differences in instrumental color perceptible by the human eye [26]). ^2^ Mean of the different replicates for each dietary treatment (n = 5 for color parameters and n = 30 for sensory scores). ^3^ Standard error of the mean. ^4^*p* values obtained from ANOVA (n = 25 for color parameters and n = 150 for sensory scores) of fresh or refrigerated fillets. Values in bold were significant (*p* < 0.05). The differences between dietary treatments found in fresh or refrigerated fillets with Scheffé's post hoc test were noted in the same column as a > b. ^5^ Pooled means of fresh or refrigerated fillets coming from the five dietary treatments (n = 25 for color parameters and n = 150 for sensory scores). ^6^*p* values obtained for the refrigeration (*p_refrigeration_*) and the interaction between the refrigeration time and the diet (*p_refrigeration x diet_*) obtained from multifactor ANOVA (n = 50 for color parameters and n = 300 for sensory scores). Values in bold were significant (*p* < 0.05).

## Data Availability

All data will be available upon request.

## Abbreviations

AO: Acid oil
CHP: Cumene hydroperoxide
DAG: Diacylglycerol
DHA: Docosahexaenoic acid
EPA: Eicosapentaenoic acid
FA: Fatty acid
FFA: Free fatty acid
FO: Fish oil
FOX: Ferrous oxidation-xylenol orange
GC-FID: Gas chromatography with flame ionization detector
HS-SPME-GC-MS: Headspace solid-phase microextraction coupled with gas chromatography and mass spectrometry
I: Insoluble impurities
LHP: Lipid hydroperoxide
M: Moisture and volatile matter
MAG: Monoacylglycerol
MDA: Malondialdehyde
MIU: Moisture and volatile matter + insoluble impurities + unsaponifiable matter
MUFA: Monounsaturated fatty acid
n-3 LC-PUFA: n-3 long-chain polyunsaturated fatty acid
ND: Not detected
OPO: Crude olive pomace oil
OPAO: Olive pomace acid oil
PUFA: Polyunsaturated fatty acid
SAO: Soybean-sunflower (55 : 45, w/w) acid oil
SFA: Saturated fatty acid
SO: Crude soybean oil
T: Tocopherol
T3: Tocotrienol
TAG: Triacylglycerol
TBA: Thiobarbituric acid
U: Unsaponifiable matter

## Supplementary Materials

Supplementary Materials The supplementary material details the analytical methods applied to diets to determine fatty acid composition, tocopherol and tocotrienol content and lipid class composition, and the complete FA profile of fish fillets, including minor FA (Table S1).
