# Supplementary material for: Effect of Feeding Acid Oils on European Seabass Fillet Lipid Composition, Oxidative Stability, Color, and Sensory Acceptance
Source: Aquac Nutr. 2023 Jan 6;2023:6415693. doi: 10.1155/2023/6415693 (PMC9973161; doi:10.1155/2023/6415693)
Supplement: Supplementary Materials — The supplementary material details the analytical methods applied to diets to determine fatty acid composition, tocopherol and tocotrienol content and lipid class composition, and the complete FA profile of fish fillets, including minor FA (Table S1). [file 6415693.f1.pdf]

Effect of feeding acid oils on European seabass fillet lipid composition, oxidative stability, color and sensory acceptance – Supplementary Material

Paula Albendea[a,b], Alba Tres[a,b,*], Magdalena Rafecas[a,c], Stefania Vichi[a,b], Roser Sala[d], Francesc Guardiola[a,b]. Aquaculture Nutrition, Article ID 6415693.

[a]Nutrition, Food Science and Gastronomy Department-XIA, Campus de l'Alimentació Torribera, Facultat de Farmàcia i Ciències de l'Alimentació, Universitat de Barcelona. Av Prat de la Riba, 171. 08921, Santa Coloma de Gramenet, Spain

[b]Institut de Recerca en Nutrició i Seguretat Alimentària (INSA-UB), Universitat de Barcelona. Av Prat de la Riba, 171. 08921, Santa Coloma de Gramenet, Spain

[c]Nutrition, Food Science and Gastronomy Department-XIA, Facultat de Farmàcia i Ciències de l'Alimentació, Universitat de Barcelona. Av Joan XXIII, 27-31. 08028, Barcelona, Spain

[d]Animal Nutrition and Welfare Service (SNiBA), Animal and Food Science Department, Facultat de Veterinària, Universitat Autònoma de Barcelona, Travessera dels Turons. 08193, Bellaterra, Spain

Corresponding author: Alba Tres, atres@ub.edu. Phone number: +34934020916

Determination of fatty acid composition of experimental diets

Lipids were extracted from 2 g of feed by using diethyl ether according to the Soxtec extraction method 2003.05 of the AOAC international [1]. After extraction, the solvent was not evaporated to dryness (up to approx. 1 mL) to prevent lipid oxidation and more diethyl ether was added to transfer the lipid extract from Soxtec vessels to two glass test tubes with screw cap (approximately half of the lipid extract to each tube). Diethyl ether was completely evaporated from tubes under a nitrogen stream at 30 °C in a block heater and the lipid extract was stored at −20 °C until the analyses. One tube was used for the determination of the fatty acid (FA) composition and the other to analyze the lipid class composition, as described below. For FA determination, the lipid extract was submitted to a double methylation in methanolic medium and FA methyl esters were determined by GC-FID [2] and quantified by peak area normalization.

Determination of tocopherol and tocotrienol content of experimental diets

The tocopherol (T) and tocotrienol (T3) determination was adapted from Bou et al. [3]. Briefly, 1.5 g of feed were homogenized using a high-speed homogenizer (Model PT 3100 Polytron, Kinematica, Lucerne, Switzerland) at 20,000 rpm for 30 s. After saponification, the non-saponifiable fraction was extracted with petroleum ether and filtered through a 0.45-μm Teflon membrane. After complete evaporation of the petroleum ether under a nitrogen stream at 30°C in a block heater, the residue was redissolved in an exact volume of 99% n-hexane and injected into the HPLC system. HPLC was performed as explained by Aleman et al. [4] and a 1260 Infinity II Fluorescence Detector (Agilent Technologies, Santa Clara, CA, USA) was used with the excitation and emission wavelengths set at 290 and 320 nm, respectively. Calibration curves were prepared for each T, using a set of T standards (α-, β-, γ- and δ-T) from Calbiochem® (Merck KGaA, Darmstadt, Germany). The content of α-, β-, γ- and δ-T3 was calculated by applying the calibration curve obtained for the corresponding T analogue.

Determination of lipid class composition of experimental diets

The lipid extraction of the feeds was performed by the Soxtec method as described above. The lipid extract was dissolved in 2 mL of tetrahydrofuran (HPLC grade from Scharlau, Sentmenat, Spain) and a 1:2 (v/v) dilution was made to obtain a lipid concentration of ≈ 15 mg/mL. Then, the percentages of triacyclglycerols, diacylglycerols, monoacylglycerols and free FA were determined by size molecular exclusion chromatography (HPLC-RID) as described by Varona et al. [2] and quantified by peak area normalization.

Table S1

Complete fatty acid profile of fresh fillets coming from fish fed with the five experimental diets.

**FA (%)**	FO[1]	SO[1]	SAO[1]	OPO[1]	OPAO[1]	SEM[2]	*p*[3]
C14:0	2.5^{a}	1.5^{c}	1.7^{b}	1.6^{bc}	1.6^{bc}	0.026	**< 0.001**
C16:0	19.2^{a}	16.4^{bc}	16.4^{bc}	16.6^{b}	16.0^{c}	0.130	**< 0.001**
C17:0	0.6^{a}	0.3^{b}	0.3^{b}	0.3^{b}	0.3^{b}	0.013	**< 0.001**
C18:0	4.7^{a}	4.6^{a}	4.3^{b}	4.0^{c}	3.9^{c}	0.053	**< 0.001**
C20:0	0.3^{ab}	0.3^{ab}	0.2^{ab}	0.3^{a}	0.2^{b}	0.006	**0.009**
C22:0	0.2^{ab}	0.2^{a}	0.2^{a}	0.1^{c}	0.2^{bc}	0.007	**< 0.001**
SFA	27.5^{a}	23.2^{b}	23.2^{b}	22.9^{bc}	22.3^{c}	0.187	**< 0.001**
C16:1 n-9	0.6^{a}	0.4^{c}	0.4^{c}	0.5^{b}	0.5^{b}	0.006	**< 0.001**
C16:1 n-7	4.0^{a}	2.3^{c}	2.5^{bc}	2.6^{b}	2.7^{b}	0.053	**< 0.001**
C18:1 n-9	25.8^{d}	27.8^{d}	30.6^{c}	43.3^{a}	40.6^{b}	0.538	**< 0.001**
C18:1 n-7	1.8^{a}	1.6^{ab}	1.4^{ab}	1.2^{b}	1.6^{ab}	0.118	**0.022**
C20:1 n-9	1.9^{a}	1.4^{d}	1.5^{cd}	1.6^{b}	1.6^{bc}	0.027	**< 0.001**
MUFA	34.0^{bc}	33.4^{c}	36.5^{b}	49.2^{a}	47.0^{a}	0.560	**< 0.001**
C18:2 n-6	11.4^{e}	26.2^{a}	23.7^{b}	12.9^{d}	15.1^{c}	0.310	**< 0.001**
C18:3 n-6	0.2^{c}	0.3^{a}	0.3^{a}	0.2^{c}	0.2^{b}	0.006	**< 0.001**
C20:2 n-6	0.7^{c}	1.0^{a}	0.9^{b}	0.5^{d}	0.5^{d}	0.018	**< 0.001**
C20:4 n-6	1.7^{a}	0.7^{b}	0.8^{b}	0.8^{b}	0.8^{b}	0.024	**< 0.001**
n-6 PUFA	13.9^{d}	28.3^{a}	25.6^{b}	14.4^{d}	16.6^{c}	0.314	**< 0.001**
C18:3 n-3	2.0^{c}	3.8^{a}	2.6^{b}	1.9^{d}	1.9^{cd}	0.028	**< 0.001**
C20:3 n-3	0.9^{a}	0.6^{b}	0.6^{b}	0.6^{b}	0.6^{b}	0.017	**< 0.001**
C20:5 n-3	5.3^{a}	2.7^{c}	3.0^{b}	2.9^{bc}	3.0^{b}	0.055	**< 0.001**
C22:6 n-3	16.1^{a}	7.7^{b}	8.2^{b}	7.9^{b}	8.1^{b}	0.293	**< 0.001**
n-3 PUFA	24.3^{a}	14.8^{b}	14.4^{b}	13.2^{b}	13.7^{b}	0.331	**< 0.001**
Total PUFA	38.2^{b}	43.0^{a}	40.0^{b}	27.6^{d}	30.3^{c}	0.483	**< 0.001**

Abbreviations: FO, fish oil diet; SO, crude soybean oil diet; SAO, soybean-sunflower acid oil diet; OPO, crude olive pomace oil diet; and OPAO, olive pomace acid oil diet; FA, fatty acid; SFA, saturated fatty acids (sum of C14:0, C16:0, C17:0, C18:0, C20:0 and C22:0); MUFA, monounsaturated fatty acids (sum of C16:1 n-9, C16:1 n-7, C18:1 n-9, C18:1 n-7 and C20:1 n-9); PUFA, polyunsaturated fatty acids (n-6 PUFA: sum of C18:2 n-6, C18:3 n-6, C20:2 n-6 and C20:4 n-6; n-3 PUFA: sum of C18:3 n-3, C20:3 n-3, C20:5 n-3 and C22:6 n-3; Total PUFA: sum of n-3 PUFA and n-6 PUFA.

[1] Data were expressed as the mean of the five replicates from each dietary treatment (n = 5).

[2] Standard error of the mean.

[3] *p* values obtained by ANOVA (n = 25). Values in bold were significant ($p < 0.05$). Differences between dietary treatments found with Scheffé's post hoc test were noted in the same row as a > b > c > d > e.

References

[1] AOAC International, "AOAC Official Method 2003.05. Crude Fat in Feeds, Cereal Grains, and Forages", in *Official Methods of Analysis of AOAC International*, *Chapter 4 - Animal Feed*, pp.41–43. AOAC International, Rockville, MD, 21th edition, 2019.

[2] E. Varona, A. Tres, M. Rafecas, S. Vichi, A. C. Barroeta, and F. Guardiola, “Methods to determine the quality of acid oils and fatty acid distillates used in animal feeding,” *MethodsX*, vol. 8, article 101334, 2021, doi: 10.1016/j.mex.2021.101334.

[3] R. Bou, F. Guardiola, A. Tres, A. C. Barroeta, and R. Codony, “Effect of dietary fish oil, α-tocopheryl acetate, and zinc supplementation on the composition and consumer acceptability of chicken meat,” *Poultry Science*, vol. 83, no. 2, pp. 282–292, 2004, doi: 10.1093/ps/83.2.282.

[4] M. Aleman, C. D. Nuchi, R. Bou, A. Tres, J. Polo, F. Guardiola, and R. Codony, “Effectiveness of antioxidants in preventing oxidation of palm oil enriched with heme iron: A model for iron fortification in baked products,” *European Journal Lipid Science and Technology*, vol. 112, no. 7, pp. 761–769, 2010, doi: 10.1002/ejlt.200900220.
